# Prevalence and distribution of HPV genotypes among HIV-infected women in Zambia

**DOI:** 10.1038/sj.bjc.6603737

**Published:** 2007-04-17

**Authors:** V V Sahasrabuddhe, M H Mwanahamuntu, S H Vermund, W K Huh, M D Lyon, J S A Stringer, G P Parham

**Affiliations:** 1 Vanderbilt University, 2215 Garland Avenue, 319 Light Hall, Nashville, TN 37232, USA; 2 University Teaching Hospital, Nationalist Road, Lusaka, Zambia; 3 Center for Infectious Disease Research in Zambia, Northmead, Lusaka, Zambia; 4 University of Alabama at Birmingham, 908-20th Street South, Birmingham, AL 35294, USA

**Keywords:** human immunodeficiency virus, human papillomavirus, cervical cancer, HPV types, Zambia

## Abstract

We screened 145 HIV-infected non-pregnant women at a tertiary care centre in Lusaka, Zambia. Liquid-based cytology and human papillomavirus (HPV) genotyping with PGMY09/11 biotinylated primers (Roche Linear Array® HPV genotyping test) maximised sensitivity of cytology and HPV assessments. Among high-risk (HR) types, HPV 52 (37.2%), 58 (24.1%) and 53 (20.7%) were more common overall than HPV 16 (17.2%) and 18 (13.1%) in women with high-grade squamous intraepithelial lesions or squamous cell carcinoma (SCC) on cytology. High-risk HPV types were more likely to be present in women with CD4^+^ cell counts <200 *μ* l^−1^ (odds ratios (OR): 4.9, 95% confidence intervals (CI): 1.4–16.7, *P*=0.01) and in women with high-grade or severe cervical cytological abnormalities (OR: 8.0, 95% CI: 1.7–37.4, *P*=0.008). Human papillomavirus diversity in high-grade lesions and SCC on cytology suggests that HPV 16- and 18-based vaccines may not be adequately polyvalent to induce protective immunity in this population.

Increasing numbers of HIV-infected women in sub-Saharan African settings like Zambia are now accessing life-prolonging antiretroviral therapy (ART) (Stringer *et al*, 2006). The consequently increasing lifespan may increase their risk of such malignancies as cervical cancer (Strickler *et al*, 2005). In a cross-sectional study among HIV-infected women seeking ART in Lusaka, Zambia, we reported a 79% prevalence of high-grade squamous intraepithelial lesions (HSIL) of the cervix (Parham *et al*, 2006). The presence of high-risk (HR) human papillomavirus (HPV) types was independently associated with high-grade cytological lesions (adjusted odds ratios (OR): 12.4, 95% confidence intervals (CI): 2.6–58.1, *P*=0.02). We were able to study HPV genotypes on stored samples from these women.

## MATERIALS AND METHODS

We enrolled 150 consecutive, consenting, non-pregnant HIV-infected Zambian women seeking HIV/AIDS treatment and care at the University Teaching Hospital, the largest tertiary-level care centre in the capital of Lusaka. All women provided written, informed consent and the study was approved by the Research Ethics Committee of the University of Zambia and the Institutional Review Board of the University of Alabama at Birmingham. Participants underwent a complete physical and gynaecological evaluation and cervical samples were collected using Ayre's spatula for the ectocervix and cytobrush for the endocervix. Samples were stored in vials containing PreservCyt® transport medium (Cytyc Corporation, Marlborough, MA, USA) at room temperature (37°C) for <2 weeks before being batch transported for cytological analysis.

Slides prepared from the PreservCyt® vials were analysed by the liquid-based ThinPrep® cytological imaging system (Cytyc Corporation, Marlborough, MA, USA). All slides were initially screened by a senior cytotechnologist and all abnormal results and 10% of negatives were subsequently reviewed by a board-certified cytopathologist. The cytology results were classified and reported as no abnormality detected (normal), atypical squamous cells of undetermined significance (ASCUS), low-grade squamous intraepithelial lesions (LSIL), HSIL and suspicious for squamous cell carcinoma (SCC).

We performed HPV typing by polymerase chain reaction (PCR)-based amplification of target DNA using the Linear Array® HPV genotyping test (LA-HPV) (Roche Molecular Systems, Pleasanton, CA, USA), an enhanced and commercialised version of the PGMY line blot assay (PGMY-LB) (Coutlee *et al*, 2002, 2006; Kornegay *et al*, 2003). The pool of consensus L1 PGMY09/11 primers used in this assay is designed to amplify HPV-DNA from 37 genotypes. These include HR HPV types (16, 18, 26, 31, 33, 35, 39, 45, 51, 52, 53, 56, 58, 59, 66, 68, 73, 82), low risk (LR) types (6, 11, 40, 42, 54, 55, 61, 70, 72, 81, and CP6108), and types with unknown risk (UR) (62, 64, 67, 69, 71, 83, 84, and IS39). Human papillomavirus types 26, 53, and 66 are usually classified as ‘probable’ HR types (Munoz *et al*, 2003), but were grouped with other HR types in this analysis to simplify interpretation.

All participants underwent visual inspection of the cervix with acetic acid and diagnostic colposcopy during the same clinic visit. A CD4^+^ cell enumeration was performed with a Beckman Coulter Epics XL-MCL 4-color Flow Cytometer (Beckman Coulter Inc. Miami, FL, USA) during the same visit unless a result was available within 3 weeks of the visit date from our rigorously certified laboratory (through the quality assurance programme of the HIV Prevention Trials Network of the National Institute of Allergy and Infectious Diseases, USA).

Using unconditional logistic regression to calculate prevalence OR and 95% CIs, we compared the type-specific HPV prevalence in HIV-infected women with CD4^+^ cell counts <200 *μ* l^−1^ against those of women with CD4^+^ cell counts ⩾200 *μ*l^−1^, the threshold to make clinical decisions for initiating ART in persons without symptoms in Zambia. Similarly, we calculated OR and 95% CIs for comparing the type-specific HPV prevalence in women with HSIL or SCC on cervical cytology and those with lower grade (ASCUS, LSIL) or no cytologic abnormalities. Statistical analysis was carried out using SPSS 14.0 for Windows™ (SPSS Inc, Chicago, IL, USA). We restricted our analysis to 145 participants with complete data both on CD4^+^ cell counts and individual HPV genotypes.

## RESULTS

The mean and median ages of these 145 participants were 36.2 years (s.d.: ±6.3) and 36 years (IQR: 31–41), respectively. About a third (54 out of 145, 37.2%) were married and cohabiting with their husband, just under a half were educated beyond high school (70 out of 145, 48.3%) and a majority (84 out of 138, 57.9%) reported a family income of less than 500 000 Zambian kwacha (∼US$110) per month. Little over a third (54 out of 144, 37.2%) reported their age of first sexual intercourse as less than 18 years, and 116 out of 145 (80%) reported between one and five lifetime sexual partners.

Nine (6.2%) women had cytologic results within normal limits. Atypical squamous cells of undetermined significance was reported in 25 (17.2%), LSIL in 34 (23.5%), HSIL in 49 (33.8%), and SCC in 28 (19.3%) participants. Thus 93.8% of these HIV-infected Zambian women seeking medical care had abnormal Pap smears, 76.6% had SIL or SCC, and 53.1% had high-grade SIL or SCC.

Mean and median CD4^+^ lymphocyte counts were 208 *μ* l^−1^ (s.d.:±177.5) and 165 *μ* l^−1^ (IQR: 85–299) respectively. Almost two thirds of the participants (*n*=91, 62.7%) had a CD4^+^ count <200 *μ* l^−1^, 43 (29.6%) had CD4^+^ counts between 200–499 *μ* l^−1^ and 11 (7.5%) had CD4^+^ cell counts ⩾500 *μ* l^−1^. Validation of results was confirmed with appropriate negative and positive controls for PCR amplification and *β*-globin gene detection. Human papillomavirus DNA was detected in 141 out of 145 (97.2%) participants, while four (2.8%) women had no evidence of cervicovaginal HPV DNA. High-risk HPV was detected in 131 (90.3%) women. We found a single HPV genotype in 14 (9.7%) women and multiple (⩾2) HPV genotypes in 127 (87.5%) women. The median number of HPV types per woman was 4 (IQR: 2–6), while the mean was 4.4 (s.d.: ±2.8). Of the 37 HPV types identifiable by the LA-HPV test, all except type 64 were detected in the 145 samples. The test identified 656 distinct HPV infections in 145 women, of which 350 (53.3%) were HR-HPV while 306 (46.6%) were LR or UR-HPV types. None of the women carried a HR-HPV type exclusively (i.e. there was always a LR/UR-HPV present if a HR-HPV was present). Among the HR types, HPV 52 was found to be the most prevalent (37.2%). However, the probe for detection of HPV-52 amplicons in the LA-HPV test is a cross-reactive probe that also hybridises with types 33, 35, and 58. It was observed that at least one of these three HR-HPV types was present in 54 (37.2%) samples along with HPV 52, while the latter was present exclusively in 11 (7.6%) samples. Human papillomavirus 58 was present in 35 (24.1%) and HPV 53 in 30 (20.7%) samples while types 16, 35 and 45 were equally common (17.2%). Human papillomavirus type 18 (13.1%) was rank-ordered 10th among high risk types.

Among the 28 women with SCC on cervical cytology, at least one HR-HPV type was present in all women while multiple (⩾2) genotypes were present in 22 (78.6%) women. Human papillomavirus type 52 (mixed probe as described above) was the most prevalent in 13 (46.4%), while types 58 and 16 were present in 10 (35.7%). The other HR types in decreasing order of their prevalence in women with SCC on cytology were HPV types 35 (28.6%), 53 (25%), 31 and 51 (21.4% each), 18 and 45 (17.9% each), 33, 56, 59, and 68 (14.3% each), 39, 36, and 66 (10.7% each), and 73 (3.6%). High-risk type 82 was not detected with SCC. Among LR or UR HPV types, types 62, 61, 70, and 84 had a prevalence of greater than 15% in cases with SCC on cytology but none of these types were present exclusively without the concurrent presence of a HR-HPV type.

High-risk human papillomavirus types were more likely to be present with CD4^+^ counts <200 *μ* l^−1^ than with higher counts (OR: 4.9, 95% CI: 1.4–16.7, *P*=0.01). Barring HPV 16 and 39, all HR types showed greater prevalence in women with CD4^+^ counts <200 *μ* l^−1^ but only type 51 achieved statistical significance (*P*=0.05), given our sample size of 145. Among HPV-types of UR, HPV 71 was more likely to be present in women with CD4^+^ counts <200 *μ* l^−1^ (*P*=0.04; Table 1).

The prevalence of HR-HPV types was higher in women with HSIL or SCC on cervical cytology (OR: 8.0, 95% CI: 1.7–37.4, *P*=0.008). High-risk human papillomavirus 58 and 31 were significantly more likely in women with HSIL or SCC (*P*=0.01 and 0.02 respectively). No other high, low, or UR type was significantly higher in prevalence in women with HSIL or SCC than in other women (Table 1).

## DISCUSSION

The high prevalence of HPV-DNA in our study (97.2% for any HPV and 90.3% for any HR-HPV) is one of the highest reported among an HIV-infected (or any other) population of women worldwide. Also, HPV types 16 and 18 were not the most prevalent HR-HPV types and a relatively high frequency of HPV types 52, 58, 35, 53, 31, 51, and 45 was observed in women who had HSIL lesions or SCC on cytology. This finding has implications in the eventual implementation of prophylactic HPV vaccines based on HR-HPV types 16 and 18 (Steinbrook, 2006). Our results add to those reported in a recent meta-analysis of HPV types among HIV-infected women (Clifford *et al*, 2006) and the recent international data which indicate the increased preponderance of HPV types other than 16 or 18 among HIV-infected women (Levi *et al*, 2002; Bollen *et al*, 2006; Didelot-Rousseau *et al*, 2006; Luque *et al*, 2006). If cross-reacting immunity is not induced across viral types by existing vaccines, this will limit their efficacy in immunosuppressed women in developing countries whose dominant HR-HPV types may be other than 16 and 18.

We noted that the majority (87.3%) of participants carried multiple HPV genotypes and that the mean number of HR types increased with increasing immunosuppression. Comparable studies using PCR-based detection of HPV in HIV-infected women have reported 12–79% of study participants with multiple HPV types (Levi *et al*, 2002; Moscicki *et al*, 2004; Bollen *et al*, 2006; Didelot-Rousseau *et al*, 2006; Hawes *et al*, 2006; Luque *et al*, 2006). This high prevalence may reflect the immune impairment by HIV that fails to clear HPV, leading to chronic HPV infection. It may also reflect HR sexual practices of some women as in the case of drug users and/or sex workers (Vermund *et al*, 1991; Shah *et al*, 1997). If HPV replication is more efficient in an immunocompromised host, the resulting higher viral load will make HPV detection easier and persistence more likely. A higher prevalence of multiple HPV genotypes can result from HIV-induced upregulation and persistence of HPV (Vernon *et al*, 1993; Palefsky *et al*, 1999; Moscicki *et al*, 2004) or from continued sexual exposure to novel HPV types during periods of severe immunosuppression. Not surprisingly, we noted no significant association between LR/UR HPV types and lower CD4^+^ cell counts in our participants, nor any association between LR/UR HPV types and presence of HSIL or severe abnormalities.

The PGMY09/11 primers used in the LA-HPV test have been validated for reproducibility and accuracy in diverse populations (Kornegay *et al*, 2003; Coutlee *et al*, 2006). The high-HPV prevalence in our study reflects both the very high sensitivity of the LA-HPV test as well as the extraordinary risk faced by Zambian women living with HIV. The differences in prevalence and diversity of HPV genotypes in HIV-infected women from different geographic origins may be due to their differing behavioural, nutritional, and socioeconomic characteristics or to male factors, as much as to the immunological status of participants. These factors need investigation in larger prospective studies around the world and especially in sub-Saharan Africa where both HIV and cervical cancer rates are high.

The results reinforce the importance of ensuring adequate cervical cancer screening services for HIV-infected women in resource-limited settings like Zambia. They are important given the recent availability of prophylactic HPV vaccines. Considering the diversity of HPV types found, HPV vaccine constructs with polyvalency would be needed for primary prevention of cervical cancer among HR women in developing country settings.

## Figures and Tables

**Table 1:** Associations between prevalent human papillomavirus (HPV) types and CD4^+^ lymphocyte counts and cervical cytological abnormalities among HIV-infected Zambian women

		**CD4^+^ cell count <200/*μ*l (*n*=91)**			**HSIL or SCC on cytology (*n*=77)**		
**HPV-type categories**	***N* (% of 145)**	**HPV +ve**	**OR (95% CI) (compared to CD4^+^ ⩾200/*μ*l)**	***P*-value**	**HPV +ve**	**OR (95% CI) (Compared to LSIL or less severe lesions on cytology)**	***P*-value**
*Presence of various categories of HPV types*							
Any HR	131(90.3%)	87	4.9 (1.4–16.7)	*0.01*	75	8.0 (1.7–37.4)	*0.008*
Any LR	87 (60%)	57	1.3 (0.67–2.7)	0.4	45	0.87 (0.45–1.7)	0.7
Any UR	87 (60%)	60	1.9 (0.97–3.9)	0.06	45	0.87 (0.45–1.7)	0.7
Multiple	127 (87.6%)	83	1.4 (0.46–4.3)	0.5	71	1.7 (0.55–5.2)	0.4
							
*Presence of individual high-risk HPV types (in order of decreasing prevalence)*							
HPV 52^a^	54 (37.2%)	39	1.9 (0.94–4.0)	0.07	32	1.5 (0.75–2.9)	0.3
HPV 58	35 (24.1%)	24	1.4 (0.62–3.1)	0.4	25	2.8 (1.2–6.3)	*0.02*
HPV 53	30 (20.7%)	20	1.2 (0.53–2.9)	0.6	17	1.2 (0.53–2.7)	0.7
HPV 16	25 (17.2%)	14	0.71 (0.27–1.9)	0.4	17	2.1 (0.85–5.3)	0.1
HPV 35	25 (17.2%)	18	1.7 (0.64–4.3)	0.3	14	1.1 (0.48–2.7)	0.8
HPV 45	25 (17.2%)	19	2.1 (0.73–6.4)	0.1	17	2.1 (0.85–5.3)	0.1
HPV 51	22 (15.2%)	18	3.1 (0.98–9.6)	*0.05*	15	2.1 (0.80–5.5)	0.1
HPV 66	21 (14.5%)	14	1.2 (0.46–3.2)	0.7	9	0.62 (0.24–1.6)	0.3
HPV 31	21 (14.5%)	14	1.2 (0.45–3.2)	0.7	16	3.3 (1.1–9.6)	*0.03*
HPV 68	20 (13.8%)	15	1.9 (0.66–5.7)	0.2	11	1.1 (0.42–2.8)	0.9
HPV 18	19 (13.1%)	12	1.0 (0.34–3.1)	0.97	10	0.98 (0.37–2.6)	0.96
HPV 39	18 (12.4%)	15	3.4 (0.92–12.8)	0.07	11	1.4 (0.53–4.0)	0.5
HPV 56	18 (12.4%)	13	1.6 (0.55–4.9)	0.4	11	1.4 (0.53–4.0)	0.5
HPV 33	12 (8.3%)	10	3.2 (0.67–15.2)	0.1	8	1.8 (0.53–6.5)	0.3
HPV 59	12 (8.3%)	5	0.39 (0.12–1.3)	0.1	5	0.60 (0.18–2.0)	0.4
HPV 52^b^	11 (7.6%)	8	1.6 (0.41–6.5)	0.5	4	0.48 (0.13–1.7)	0.3
HPV 73	10 (6.9%)	7	1.4 (0.35–5.7)	0.6	4	0.57 (0.15–2.1)	0.4
HPV 26	9 (6.2%)	7	2.2 (0.43–10.8)	0.4	7	3.3 (0.66–16.5)	0.1
HPV 82	7 (4.8%)	5	1.5 (0.28–8.1)	0.6	5	2.3 (0.43–12.2)	0.3
							
*Presence of individual low-risk HPV types (in order of decreasing prevalence)*							
HPV 61	39 (26.9%)	25	1.1 (0.50–2.3)	0.8	19	0.79 (0.38–1.6)	0.5
HPV 81	26 (17.9%)	17	1.1 (0.47–2.8)	0.8	14	1.0 (0.44–2.4)	0.9
CP 6108	20 (13.8%)	14	1.4 (0.52–4.0)	0.5	9	0.69 (0.27–1.8)	0.4
HPV 70	18 (12.4%)	11	0.92 (0.33–2.5)	0.9	10	1.12 (0.41–3.0)	0.8
HPV 42	17 (11.7%)	11	1.1 (0.38–3.2)	0.9	8	0.76 (0.28–2.1)	0.6
HPV 72	13 (8.9%)	11	3.6 (0.76–16.8)	0.1	6	0.74 (0.23–2.3)	0.6
HPV 54	12 (8.3%)	8	1.2 (0.34–4.2)	0.8	5	0.60 (0.18–2.0)	0.4
HPV 40	9 (6.2%)	5	0.72 (0.18–2.8)	0.7	4	0.69 (0.18–2.7)	0.6
HPV 6	5 (3.4%)	5	1 (—)	—	3	1.4 (0.22–8.2)	0.8
HPV 11	1 (0.7%)	1	1 (—)	—	0	—	—
							
*Presence of individual unknown-risk HPV types (in order of decreasing prevalence)*							
HPV 62	37 (25.5%)	24	1.1 (0.52–2.5)	0.8	24	1.9 (0.88–4.1)	0.1
HPV 84	26 (17.9%)	18	1.4 (0.57–3.5)	0.5	13	0.86 (0.37–2.0)	0.7
HPV 71	23 (15.9%)	19	3.3 (1.1–10.3)	*0.04*	14	1.5 (0.59–3.6)	0.4
HPV 83	16 (11.0%)	11	1.3 (0.44–4.1)	0.6	6	0.49 (0.17–1.4)	0.2
HPV 55	14 (9.7%)	12	3.9 (0.85–18.4)	0.08	6	0.63 (0.21–1.9)	0.4
HPV 67	9 (6.2%)	6	1.2 (0.29–5.0)	0.8	5	1.1 (0.29–4.3)	0.9
IS 39	8 (5.5%)	5	0.98 (0.23–4.3)	0.98	4	0.88 (0.21–3.6)	0.9
HPV 69	2 (1.4%)	1	0.59 (0.03–9.6)	0.7	1	0.88 (0.05–14.4)	0.9
HPV 64	0 (0%)	0	—	—	0	—	—

HSIL=high-grade squamous intraepithelial lesions; SCC=squamous cell carcinoma; LSIL=low-grade squamous intraepithelial lesions; HR=high-risk HPV types; LR=low-risk HPV types; UR: HPV types of unknown risk.

^a^and ^b^: Probe for detection of HPV 52 cross reacts with HPV 33, 35, and 58. Thus, ^a^is HPV 52 with HPV type 33, 35, and/or 58 coinfection while ^b^is HPV 52 present without any coinfection.
